# Comparative Analysis and Distribution of Omega-3 lcPUFA Biosynthesis Genes in Marine Molluscs

**DOI:** 10.1371/journal.pone.0136301

**Published:** 2015-08-26

**Authors:** Joachim M. Surm, Peter J. Prentis, Ana Pavasovic

**Affiliations:** 1 School of Biomedical Sciences, Faculty of Health, Queensland University of Technology, 2 George Street, Brisbane, Qld 4000, Australia; 2 School of Earth, Environmental and Biological Sciences, Science and Engineering Faculty, Queensland University of Technology, 2 George Street, Brisbane, Qld 4000, Australia; Universiti Sains Malaysia, MALAYSIA

## Abstract

Recent research has identified marine molluscs as an excellent source of omega-3 long-chain polyunsaturated fatty acids (lcPUFAs), based on their potential for endogenous synthesis of lcPUFAs. In this study we generated a representative list of fatty acyl desaturase (Fad) and elongation of very long-chain fatty acid (Elovl) genes from major orders of Phylum Mollusca, through the interrogation of transcriptome and genome sequences, and various publicly available databases. We have identified novel and uncharacterised Fad and Elovl sequences in the following species: *Anadara trapezia*, *Nerita albicilla*, *Nerita melanotragus*, *Crassostrea gigas*, *Lottia gigantea*, *Aplysia californica*, *Loligo pealeii* and *Chlamys farreri*. Based on alignments of translated protein sequences of Fad and Elovl genes, the haeme binding motif and histidine boxes of Fad proteins, and the histidine box and seventeen important amino acids in Elovl proteins, were highly conserved. Phylogenetic analysis of aligned reference sequences was used to reconstruct the evolutionary relationships for Fad and Elovl genes separately. Multiple, well resolved clades for both the Fad and Elovl sequences were observed, suggesting that repeated rounds of gene duplication best explain the distribution of Fad and Elovl proteins across the major orders of molluscs. For Elovl sequences, one clade contained the functionally characterised Elovl5 proteins, while another clade contained proteins hypothesised to have Elovl4 function. Additional well resolved clades consisted only of uncharacterised Elovl sequences. One clade from the Fad phylogeny contained only uncharacterised proteins, while the other clade contained functionally characterised delta-5 desaturase proteins. The discovery of an uncharacterised Fad clade is particularly interesting as these divergent proteins may have novel functions. Overall, this paper presents a number of novel Fad and Elovl genes suggesting that many mollusc groups possess most of the required enzymes for the synthesis of lcPUFAs.

## Introduction

Most vertebrates are considered inefficient at producing long-chain polyunsaturated fatty acids (lcPUFAs), such as omega-3, which makes them essential dietary requirements. In particular, omega-3 lcPUFAs are widely recognised for their role in neurological development, immune response and cardiac and circulatory function [1–6]. Despite some debate about the specific role of lcPUFAs in disease progression [2, 7–9], omega-3s are increasingly being used to biofortify human and animal diets. To meet this growing demand, marine molluscs have emerged as an excellent source of omega-3 lcPUFAs, not only because of their relatively high content of omega-3, but for their ability to endogenously elongate and desaturate precursor fatty acids to produce lcPUFAs [10].

Generally, lcPUFAs are synthesised by conversion of dietary PUFAs (e.g. 18-C molecule) to lcPUFAs (e.g. >20-C molecule) [11]. Specifically, PUFAs converted to lcPUFAs include linoleic acid (LA, C18:2n-6) and α-linolenic acid (ALA, C18:3n-3), with end products including omega-3 lcPUFAs such as eicosapentaenoic acid (EPA; 20:5n-3) and docosahexaenoic acid (DHA; 22:6n-3). These lcPUFAs are largely derived through the action of two key classes of enzymes; desaturases and elongases. While biosynthesis of EPA and DHA is well studied, the distribution and evolution of the genes encoding enzymes responsible for extension of the carbon chain and desaturation of bonds remain less understood. The genes that encode for the main elongases are the elongation of very long-chain fatty acid 2, 4 and 5 (*Elovl2*, *Elovl4 and Elovl5*) [12–14]. The fatty acyl desaturase (Fad) gene family encodes for desaturase enzymes, of which delta-5 and delta-6 are the most significant in desaturation of precursor PUFA molecules to lcPUFA, such as EPA and DHA. Based on the current literature, the distribution of these genes appears to be lineage specific, with different members of both classes being lost or duplicated in various taxa [15–18]. This may be explained by the apparent redundancy in function observed within the Elovl gene family. For example, both *Elovl2* and *Elovl4* elongate molecules from C-22 to C-24, in DHA synthesis [19,20]. An additional example is the failure to identify *Elovl2* in marine fish, including those with sequenced genomes [10,20], as well as the loss of *delta-5 Fad* gene in the majority of teleost species [12–14]. While the distribution of key Elovl and Fad genes remains largely unresolved in vertebrate taxa, even less information currently exists describing the homologous genes and their distribution in invertebrate species [21].

Currently, marine invertebrates such as cephalopods, bivalves and gastropods are frequently used as a dietary source of omega-3 lcPUFAs [22]. While a number of studies have demonstrated that some mollusc species are able to modify their lcPUFA content, only a few studies have investigated the endogenous Fad and Elovl genes required for the synthesis of omega-3 lcPUFAs [10]. Specifically, these include sequences of Fad and Elovl genes isolated from *Octopus vulgaris* [10,16,23,24], *Haliotis discus* [25] and *Chlamys nobilis* [26,27]. A total of nine sequences are currently available for molluscs, seven of which have been functionally characterised. One characterised Elovl gene from *O*. *vulgaris* is hypothesised to have Elovl4 activity, while the other Elovls from the *O*. *vulgaris* and *C*. *nobilis*, have Elovl5 function [10,23,27]. All characterised Fad genes in molluscs have demonstrated delta-5 desaturase activity [16,25,26]. Given the lack of sequence data for molluscs, it is therefore not surprising that no studies, to date, have investigated the phylogenetic relationship and distribution of Fad and Elovl gene families across this phylum. Recently there has been a large increase in publically available genome and transcriptome data for non-model species [28]. This increase in data and associated improvements in bioinformatics and computation has allowed the prediction and identification of functional genes in diverse taxonomic groups. Using improved gene identification techniques, this study examined the distribution of Fad and Elovl genes found in all major orders of phylum Mollusca, through the interrogation of the available transcriptome and genome sequences, as well as key protein databases. Phylogenetic analysis of this data has also been performed to elucidate the evolution of the Fad and Elovl gene families in molluscs.

## Methods

### Identification of candidate genes

To obtain a representative list of Fad and Elovl genes from the following orders of phylum Mollusca; Bivalvia, Gastropoda and Cephalopoda, we have interrogated multiple transcriptome sequences, publicly available mollusc genomes, searched the sequence read archive (SRA) for non-assembled mollusc transcriptomes, as well as retrieved the available protein sequences from UNIPROT and NCBI Non-Redundant (NR) database.

### Generating novel sequences from candidate species

The genes involved in the synthesis of omega-3 lcPUFAs were investigated in three candidate mollusc species with sequenced transcriptomes. These species include an intertidal bivalve *Anadara trapezia* (2.4 Gb of total sequence data; [29]) and two closely related, intertidal gastropod species, *Nerita albicilla* and *Nerita melanotragus* (~7 Gb of total sequence for each species; NCBI SRA accession numbers SRP056343 (*N*. *albicilla*) and SRP056344 (*N*. *melanotragus*). The two neritid species of marine snails are common to the intertidal zone in Australia [30], and are ecologically similar to other intertidal marine snails currently used as commercial sources of omega-3 in Asian countries [31]. *Anadara trapezia* is a bivalve commonly found in Australia and is closely related to *Anadara granosa*, a key aquaculture species in Malaysia and a number of other Asian countries [32]. Currently, *A*. *trapezia* is being developed for aquaculture production in Australia [33]. Briefly, the transcriptomes were sequenced on a HiSeq 2000 (Illumina, San Diego, USA) using 91-bp paired-end chemistry. High quality paired-end reads (Q > 30, ambiguous bases < 1%) were *de novo* assembled into contigs using multiple software including CLC genomics workbench v6.0.4 (CLC Inc, Aarhus, Denmark) and Trinity short read *de novo* assembler [34]. Redundant contigs and chimeric sequences were removed using CD-hit [35]. Contigs of ≥ 200bp were selected and annotated against the NCBI NR database as BLASTx queries using BLAST+ 2.2.29 software with stringency E-value of 1E-6 (this E-value was used for all BLAST searches). Functional gene ontology (GO) annotations were performed using Blast2GO software [36]. Genes of interest were identified in the transcriptomes with relevant BLASTx, GO or enzyme code annotations associated with known lcPUFA synthesis. Candidate genes were also identified using a custom generated BLAST database (CB database) created from known omega-3 lcPUFA synthesis genes in molluscs extracted from the UNIPROT database.

### Bioinformatic analysis of available genomes and genomic resources

To identify candidate Fad and Elovl genes from other mollusc taxa, the NCBI NR protein database and the UNIPROT database were interrogated using taxonomically restricted BLAST searches (molluscs (taxid: 6447)). Furthermore, the genomic and proteomic data from the largely complete draft genomes of *Crassostrea gigas* (oysterDB), *Lottia gigantea* (JGI) and *Aplysia californica* (Broad Institute) were interrogated for full-length proteins, Pfam domains [37] and GO terms corresponding to Fad and Elovl proteins. The transcriptome sequences of *Loligo pealeii*, *Chlamys farreri*, *Argopecten irradians* and *Chiton olivaceus* were also interrogated for Fad and Elovl genes. The transcriptomes of *C*. *farreri*, *A*. *irradians* and *C*. *olivaceus*, however, were only available as raw sequence reads and required bioinformatic assembly for candidate gene identification. Raw paired-end reads for these three species were downloaded from the SRA database at NCBI (*C*. *farreri* SRR653436 [38], *A*. *irradians* SRX470082 [39], *C*. *olivaceus* SRX205322 [40]). The SRA files were converted to left and right fastq files using NCBI SRA toolkit version 2.3.5, fastq dump, and assembled using Trinity *de novo* assembler software. Contigs from *L*. *pealeii*, *C*. *farreri*, *A*. *irradians* and *C*. *olivaceus* of ≥ 200bp were used as BLASTx queries against our custom BLAST database generated from known omega-3 lcPUFA synthesis genes from molluscs using the BLAST+ 2.2.29 software.

Identified candidate genes were considered full-length proteins after their open reading frames (ORFs) were determined using ORF Finder [41], all conserved domains found in these proteins were predicted using SMART database [42] searches and when BLASTp searches returned full-length alignments against known full-length proteins from other species. We further refined our sequence list to include only functional proteins, which were characterised by the presence of essential structural characteristics. These included a N-terminal cytochrome b5-like binding domain (cyt-b5; PF00173), three histidine boxes (HXXXH, HXXHH and QXXHH) located in a fatty acid desaturase domain (FA_desaturase; PF00487), and a haeme binding motif (HPGG) in Fads. The structural characteristics of Elovls included a diagnostic histidine box motif (HXXHH), a Pfam ELO domain (PF01151), the predicted trans-membrane domains and the presence of 17 highly conserved amino acid residues distributed throughout the protein (125K, 128E, 131DT, 137L, 151HH, 178N, 182H, 185MY, 188YY, 208T, 250LF, 254F) annotated from *O*. *vulgaris* Elovl5 protein (AFM93779) [43]. These structural characteristics are essential for desaturation and elongation, and therefore transcripts not containing these domains were not considered. The position and number of transmembrane domains was determined for full-length proteins using TMHMM Server v. 2.0 [44]. All full-length sequences that did not possess the specified features were considered putative pseudogenes.

To better understand the distribution of the Fad and Elovl gene families, we investigated the homology of gene sequences and exon-intron structure of these genes across multiple taxa. Local BLAST analysis, using custom Perl scripts, was performed to identify homologous protein sequences for both Fad and Elovl genes. These candidate protein sequences were used as BLASTx queries against CB database. Exon-intron structure was investigated for all candidate genes based on *C*. *gigas* and *L*. *gigantea* genome sequences in ENSEMBL Metazoa database [45].

### Phylogenetic analyses

The refined list of full-length ORFs and their translated protein sequences from mollusc species were used for phylogenetic analyses to determine the distribution of Fads and Elovls across this phylum. Initially, protein sequences were aligned in Geneious version 7.1.5 [46] using a global alignment with a Blosum62 cost matrix [47]. Aligned sequences were then manually edited in BioEdit v7.2.5 [48] to identify conserved residues and motifs using an annotated protein sequence from *C*. *nobilis* for Fad proteins (AIC34709) and from *O*. *vulgaris* for Elovl proteins (Elovl4 AIA58679, Elovl5 AFM93779).

Protein sequences for both Fad (XP_001640617) and Elovl (XP_001640727) genes, from the *Nematostella vectensis*, were used as an outgroup for both phylogenetic trees. No vertebrate sequences were included for either gene family, as previous research has shown that genes from molluscs fall outside of all vertebrate lineages [25–27]. For the Elovl phylogeny, Elovl 1, 3, 6 and 7 were excluded from this analysis as they play no role in the synthesis of the lcPUFAs EPA and DHA [27].

To infer the evolutionary history of Fad and Elovl genes, Maximum Likelihood method was performed on both nucleotide and protein sequence datasets. The full-length ORFs were aligned using M-coffee [49] and the optimal model of nucleotide evolution was determined using MEGA6 [50]. Finally, Maximum Likelihood trees were constructed with a Tamura 3-parameter substitution model with Gamma distribution and invariant sites selected for the Fad phylogeny, and a Kimura 2-parameter substitution model with Gamma distribution and invariant sites selected for the Elovl phylogeny, both undergoing 1000 bootstrap iterations. Protein sequences were aligned using M-coffee [49] and the optimal model of amino acid substitution was determined using MEGA6 [50]. Maximum Likelihood trees were then constructed using LG amino acid substitution model with Gamma distribution and invariant sites selected for both the Fad and Elovl trees and undergoing 1000 bootstrap iterations

### cDNA amplification of candidate transcripts

To confirm the reliability of our *de novo* assemblies, three candidate transcripts were validated using cDNA synthesis, cloning and sequencing from newly collected *A*. *trapezia* and *N*. *melanotragus* samples. *Anadara trapezia* were collected from seagrass beds at Wynnum, Queensland, Australia (GPS Position: 27°26&rsquo;08.7"S 153°10&rsquo;25.0"E) and *N*. *melanotragus* were collected from King’s Beach, Caloundra, Queensland, Australia (GPS Position: 153°8&rsquo;14"E, 26°48&rsquo;17"S). Sample collection has been authorised under the Fisheries Act 1994 (General Fisheries Permit), permit number: 166312. Whole organisms were snap frozen using liquid N_2_ and stored at—80°C. Tissue samples were ground in liquid N_2_, with total RNA isolated using TRIzol reagent (Life Technologies, Carlsbad, CA, USA) and cleaned up using a RNeasy Mini Kit (Qiagen, Victoria, Australia). Genomic DNA still present following RNA extraction was digested using Turbo DNA-free kit (Life Technologies, Carlsbad, CA, USA).

Primers were designed using Primer3 software with the settings from [51] to amplify the entire ORF of the candidate genes, as per Table 1. RNA samples were converted to cDNA and PCR amplified using the MyTaq One-Step RT-PCR Kit (Bioline, London, UK). All PCR conditions followed the manufacturer’s protocol except annealing temperatures, which were optimised for individual primer pairs (Table 1). PCR products were run on a 1.5% agarose gel and stained using gel red (Bioline, London, UK). Amplification of a PCR product of the correct size was considered to be the correct transcript. PCR products were purified using an Isolate II PCR and Gel Kit (Bioline, London, UK) and cloned into a plasmid using the Promega pGEM-T Easy Vector kit (Promega, Madison, WI, USA). A minimum of two clones were selected and purified using an Isolate II Plasmid minikit (Bioline, London, UK) and sequenced using the primers M13–47 and RV-M (Promega, Madison, WI, USA). Cycle sequencing was performed in duplicate and in both forward and reverse direction using the BIGDYE Terminator Cycle Sequencing kit version 3.1 (Life Technologies, Carlsbad, CA, USA). Sequencing products were cleaned up with an ethanol-EDTA precipitation and run on a 3500 Genetic Analyzer (Life Technologies, Carlsbad, CA, USA). Geneious version 7.1.5 [46] software package was used to visualise, edit and concatenate sequences for all loci. Sequences for all successfully amplified loci were compared to the *de novo* assembled contig from which the primers were designed using a global nucleotide alignment with free-end gaps, and 93% similarity cost matrix.

**Table 1:** List of primers, including annealing temperature, designed to validate Fad and Elovl genes, identified from *A*. *trapezia* and *N*. *melanotragus*.

Species	Contig name	Primer	Primer sequence	Annealing temperature (°C)
*A*. *trapezia*	AtFad	ATDF	CTGATGGCACTTCCATTGTG	54
		ATDR	TAACGACGCGCGTGTATTAG	
*N*. *melanotragus*	NmElovla	NMEF1	TGGTCGCACTATCCTGTACG	52
		NMER1	TCACAGGCCTCAGTTTGATCT	
	NmElovlb	NMEF2	GTCTACAGCGTGGGTGGTG	52
		NMER2	GCCATTTAATGCCAATGTGT	

## Results

### Identification of candidate genes

Multiple Fad and Elovl transcripts were identified in the transcriptomes of the three candidate species (Table 2). In *A*. *trapezia*, a transcript relating to a single Fad (AtFad; accession number KR154727) was identified after two separate contigs, which received the same top BLAST hit (from *C*. *gigas*; accession number EKC30965), were manually merged at a 15 bp overlapping region. The AtFad transcript (1293 bp) encoded a 432 amino acid (aa) protein, with cyt-b5 (PF00173) and FA_desaturase (PF00487) domains. No full-length Elovl proteins were found in *A*. *trapezia*. While a number of incomplete transcripts were observed, for the purposes of this manuscript, they were not considered as they did not contain an ELO domain (PF01151) or the correct number of transmembrane domains (5–7).

**Table 2:** List of candidate genes that encode putative Fad and Elovl proteins identified from *A*. *trapezia*, *N*. *albicilla* and *N*. *melanotragus* transcriptome assemblies.

Species	Gene	Contig number	Contig name	Transcript length (bp)	Full length	Protein length (aa)	Pfam domain
*A*. *trapezia*	*Fad*	CL5362.Contig1		1068	No		
		CL2092.Contig1		234	No		
		CL5362/CL2092 merged	AtFad	1293	Yes	432	Cyt-b5; FA_desaturase
*N*. *albicilla*	*Fad*	comp92028_c0_seq5	NaFada	1580	Yes	442	Cyt-b5; FA_desaturase
		comp92028_c0_seq9	NaFada	1541	Yes	442	Cyt-b5; FA_desaturase
		comp92028_c0_seq6	NaFadb	1608	Yes	432	Cyt-b5; FA_desaturase
	*Elovl*	comp87569_c0_seq1	NaElovla	1289	Yes	304	ELO
		comp69709_c1_seq1	NaElovlb	967	Yes	278	ELO
		comp84775_c1_seq1	NaElovlc	1107	Yes	292	ELO
*N*. *melanotragus*	*Fad*	comp87301_c0_seq1	NmFada	1557	Yes	432	Cyt-b5; FA_desaturase
		contig_24618	NmFada	1626	Yes	432	Cyt-b5; FA_desaturase
		comp88083_c1_seq1	NmFadb	1607	Yes	444	Cyt-b5; FA_desaturase
		contig_5699	NmFadc	1765	Yes	443	Cyt-b5; FA_desaturase
	*Elovl*	comp59448_c0_seq1	NmElovla	1372	Yes	266	ELO
		comp78993_c0_seq1	NmElovlb	1216	Yes	293	ELO
		comp84505_c0_seq1	NmElovlc	3349	Yes	306	ELO
		contig_548	NmElovld	819	Yes	261	ELO

In *N*. *albicilla*, three full-length Fad and three Elovl transcripts were identified. Both NaFada transcripts (comp92028_c0_seq5 and comp92028_c0_seq9) had identical ORFs at the nucleotide level and were considered allelic or splice variants of the same gene. The two different Fad proteins, NaFada and NaFadb, had ORFs that translated into predicted proteins of 442 and 432 aa, respectively. The three Elovl proteins were different in ORF nucleotide sequence and translated into proteins of 304 (NaElovla), 278 (NaElovlb) and 292 (NaElovlc) aa in length. All of the full-length putative proteins contained the required conserved domains (i.e. cyt-b5 and FA_desaturase for Fad, and ELO domain in Elovl transcripts) and the correct number of transmembrane domains (i.e. 3–4 for Fads and 5–7 for Elovls) for proper protein function.

The *N*. *melanotragus* transcriptome also contained multiple full-length Fad and Elovl transcripts. These included four full-length Fads (comp87301_c0_seq1, contig_24618, comp88083_c1_seq1 and contig_5699) and four Elovl transcripts (comp59448_c0_seq1, comp78993_c0_seq1, comp84505_c0_seq1 and contig_548). Of the four full-length Fads, only three were unique. The unique Fads translated into proteins of 432 (NmFada), 444 (NmFadb) and 443 (NmFadc) aa in length. The Elovl transcripts translated into 266 (NmElovla; accession number KR154728), 293 (NmElovlb; accession number KR154729), 306 (NmElovlc) and 261 (NmElovld) aa in length.

Searches of the NCBI NR protein database and the UNIPROT database using taxonomically restricted BLAST searches identified only a limited number of sequences for Fad and Elovl genes, and their corresponding proteins, in these databases. In total, five Fad and four Elovl proteins were identified from mollusc species (Table 3). Four Fad proteins were identified in *C*. *nobilis* (1 protein; AIC34709), *H*. *discus* (2 proteins; ADK38580 and ADK12703) and *O*. *vulgaris* (1 protein; AEK20864) were all functionally characterised with delta-5 desaturase activity. An additional Fad protein was also found in *Sepia officinalis* (AKE92955) that is yet to be functionally characterised. The three Elovl proteins were identified in *C*. *nobilis* (1 protein; AGW22128) and *O*. *vulgaris* (2 proteins; AFM93779 and AIA58679). Two Elovl proteins had been previously functionally characterised in *O*. *vulgaris* (AFM93779) and *C*. *nobilis* (AGW22128) protein having Elovl5 activity. An additional Elovl protein has been found in *O*. *vulgaris* (AIA58679) with preliminary data suggesting that this protein may have Elovl4 activity. An Elovl (Elovl4 function) and a Fad (delta-8 function) protein have been characterised in the bivalve *C*. *nobilis* [52], however, these sequences are currently not publicly available.

**Table 3:** List of mollusc Fad and Elovl genes, currently available in NCBI NR protein and UNIPROT databases.

Species	Gene	Definition	Protein length (aa)	Protein accession	Reference	Function
*C*. *nobilis*	*Fad*	delta-5 fatty acyl desaturase	428	AIC34709	[26]	Delta-5
	*Fads2*	delta-8 fatty acyl desaturase	436	NA	[52]	Delta-8
	*Elovl*	elongase of very long-chain fatty acids-like protein	307	AGW22128	[27]	Elovl5
	*Elovl4*	elongase of very long-chain fatty acids-like protein	308	NA	[52]	Elovl4
*H*. *discus*	*Fad1*	delta-5 fatty acid desaturase 1	438	ADK38580	[25]	Delta-5
	*Fad1*	delta-5 fatty acid desaturase 2	439	ADK12703	[25]	Delta-5
*O*. *vulgaris*	*Fad*	delta-5 fatty acyl desaturase	445	AEK20864	[16]	Delta-5
	*Elovl*	elongase of very long-chain fatty acids-like protein	294	AFM93779	[23]	Elovl5
	*Elovl4*	elongation of very long-chain fatty acids protein 4	309	AIA58679	[10]	Elovl4-like
*S*. *officinalis*	*Fad*	delta-5 fatty acyl desaturase	445	AKE92955	Unpublished	NA
	*Elovl*	elongase of very long-chain fatty acids-like protein	295	AKE92956	Unpublished	NA

Searches of the three currently available mollusc genomes, *C*. *gigas*, *L*. *gigantea* and *A*. *californica*, identified multiple full-length Fad and Elovl genes in each species (Table 4). We identified four Fads (EKC30965, EKC33620, XP_011414050 and EKC42380) and seven Elovls (EKC19804, EKC25061, EKC39214, EKC41251, XP_011450775, XP_011450777 and CGI_10028198) in the *C*. *gigas* genome sequence, however, one Elovl protein (CGI_10028198) was only identified following local BLAST searches against our CB database (Table 4). The unannotated transcript (CGI_10028198) was 930bp in length, and encoded a putative protein of 309 aa. Another identified Elovl gene (EKC19804) encoded a 524 aa protein but was cropped to 267 aa, as the first 267 aa had a significant BLAST hit to a known *O*. *vulgaris* Elovl protein, and no other supporting RNA-Seq or protein evidence for a longer protein was found. Analysis of the *L*. *gigantea* genome revealed the presence of three Fad (XP_009049968, XP_009045077, XP_009051231) and two Elovl proteins (XP_009051096, XP_009045720) (Table 4). Four full-length Fad genes (XP_005090573, XP_005090577, XP_005093182, XP_005097048) and three full-length Elovl genes (XP_005098302, XP_005106660, XP_005095683) were identified in the *A*. *californica* genome sequence (Table 4). These searches also revealed the presence of a number of partial or interrupted genes from both protein classes in the genome sequences of all species. These gene fragments would not produce functional proteins and were excluded from further analysis as they lacked relevant Pfam domains, transmembrane domains and conserved aa residues required for correct protein function.

**Table 4:** Fad and Elovl genes extracted from the currently available complete mollusc genomes of *C*. *gigas*, *A*. *californica* and *L*. *gigantea*.

Species	Gene	Protein	Sequence ID	Protein length (aa)	Accession
*C*. *gigas*	*Fad*	Fatty acid desaturase 2	*C*. *gigas* 2	433	EKC30965
		Fatty acid desaturase 2	*C*. *gigas* 1	436	EKC33620
		Fatty acid desaturase 2	*C*. *gigas* 3	433	XP_011414050
	*Elovl*	Elongation of very long-chain fatty acids protein 4	*C*. *gigas* 5	267	EKC19804
		Elongation of very long-chain fatty acids protein 4	*C*. *gigas* 3	291	EKC25061
		Elongation of very long-chain fatty acids protein 4	*C*. *gigas* 4	262	EKC39214
		Elongation of very long-chain fatty acids protein 4	*C*. *gigas* 2	269	EKC41251
		Elongation of very long-chain fatty acids protein 4-like isoform X1	*C*. *gigas* 6	298	XP_011450775
		Elongation of very long-chain fatty acids protein 4-like isoform X2	*C*. *gigas* 7	295	XP_011450777
		unannotated	*C*. *gigas* 1	309	CGI_10028198
*A*. *californica*	*Fad*	fatty acid desaturase 1-like	*A*. *californica* 3	433	XP_005090573
		fatty acid desaturase 1-like isoform X5	*A*. *californica* 4	433	XP_005090577
		fatty acid desaturase 2-like	*A*. *californica* 2	434	XP_005093182
		fatty acid desaturase 2-like	*A*. *californica* 1	432	XP_005097048
	*Elovl*	elongation of very long-chain fatty acids protein 5-like	*A*. *californica* 1	305	XP_005098302
		elongation of very long-chain fatty acids protein 4-like	*A*. *californica* 2	324	XP_005095683
		elongation of very long-chain fatty acids protein 4-like	*A*. *californica* 3	307	XP_005106660
*L*. *gigantea*	*Fad*	hypothetical protein LOTGIDRAFT_113523	*L*. *gigantea* 2	434	XP_009049968
		hypothetical protein LOTGIDRAFT_198790	*L*. *gigantea* 3	431	XP_009045077
		hypothetical protein LOTGIDRAFT_170234	*L*. *gigantea* 1	435	XP_009051231
	*Elovl*	hypothetical protein LOTGIDRAFT_214031	*L*. *gigantea* 2	288	XP_009051096
		hypothetical protein LOTGIDRAFT_199086	*L*. *gigantea* 1	283	XP_009045720

Analysis of the four other mollusc transcriptomes identified full-length transcripts only in the *L*. *pealeii* and *C*. *farreri* assemblies (Table 5). Partial transcripts were identified in both the *A*. *irradians* and *C*. *olivaceus* transcriptomes for both protein classes but no full-length genes were identified. A single Fad and two Elovl transcripts were identified in the *L*. *pealeii* assembly (Table 5). The ORF for *L*. *pealeii* Fad translated into a 445 aa protein, while the two Elovl transcripts had ORFs that translated into proteins of 296 and 322 aa. Two Fad transcripts were identified in the *C*. *farreri* transcriptome (Table 5), and their ORFs translated into 428 and 436 aa proteins. Assembly statistics for the *C*. *farreri*, *A*. *irradians* and *C*. *olivaceus* transcriptomes are presented in Table 6. The mean contig length was 615, 629 and 427bp, and N50 was 834, 900 and 506bp for *C*. *farreri*, *A*. *irradians* and *C*. *olivaceus*, respectively. The assembly metrics are within the reported range for other mollusc species [29].

**Table 5:** List of candidate genes that encode putative Fad and Elovl proteins identified from *L*. *pealeii* and *C*. *farreri* transcriptome assemblies.

Species	Gene	Contig number	Contig name	Transcript length (bp)	Full length	Protein length (aa)	Pfam domains
*L*. *pealeii*	*Fad*	VL.id87430.tr217207	*L*. *pealeii* 1	2093	Yes	445	Cyt-b5; FA_desaturase
	*Elovl*	SG.id94038.tr94065	*L*. *pealeii* 1	1221	Yes	296	ELO
	*Elovl*	VL.id104916.tr79693	*L*. *pealeii*	1246	Yes	322	ELO
*C*. *farreri*	*Fad*	comp45465_c0_seq1	*C*. *farreri* 2	1524	Yes	428	Cyt-b5; FA_desaturase
	*Fad*	comp76054_c0_seq1	*C*. *farreri* 1	1535	Yes	436	Cyt-b5; FA_desaturase

**Table 6:** Assembly summary statistics, for *C*. *farreri*, *A*. *irradians* and *C*. *olivaceus* transcriptomes using Trinity *de novo* assembler.

Species	Number of contigs	N50	Mean contig length (bp)	Longest contig (bp)	Accession SRA	Reference
*C*. *farreri*	132 529	834	615	14797	SRR653436	[38]
*A*. *irradians*	82 345	900	629	34005	SRX470082	[39]
*C*. *olivaceus*	218350	506	427	12430	SRX205322	[40]

### Phylogenetic analyses

#### Fads alignment and phylogenetic analysis

**Fig 1:**
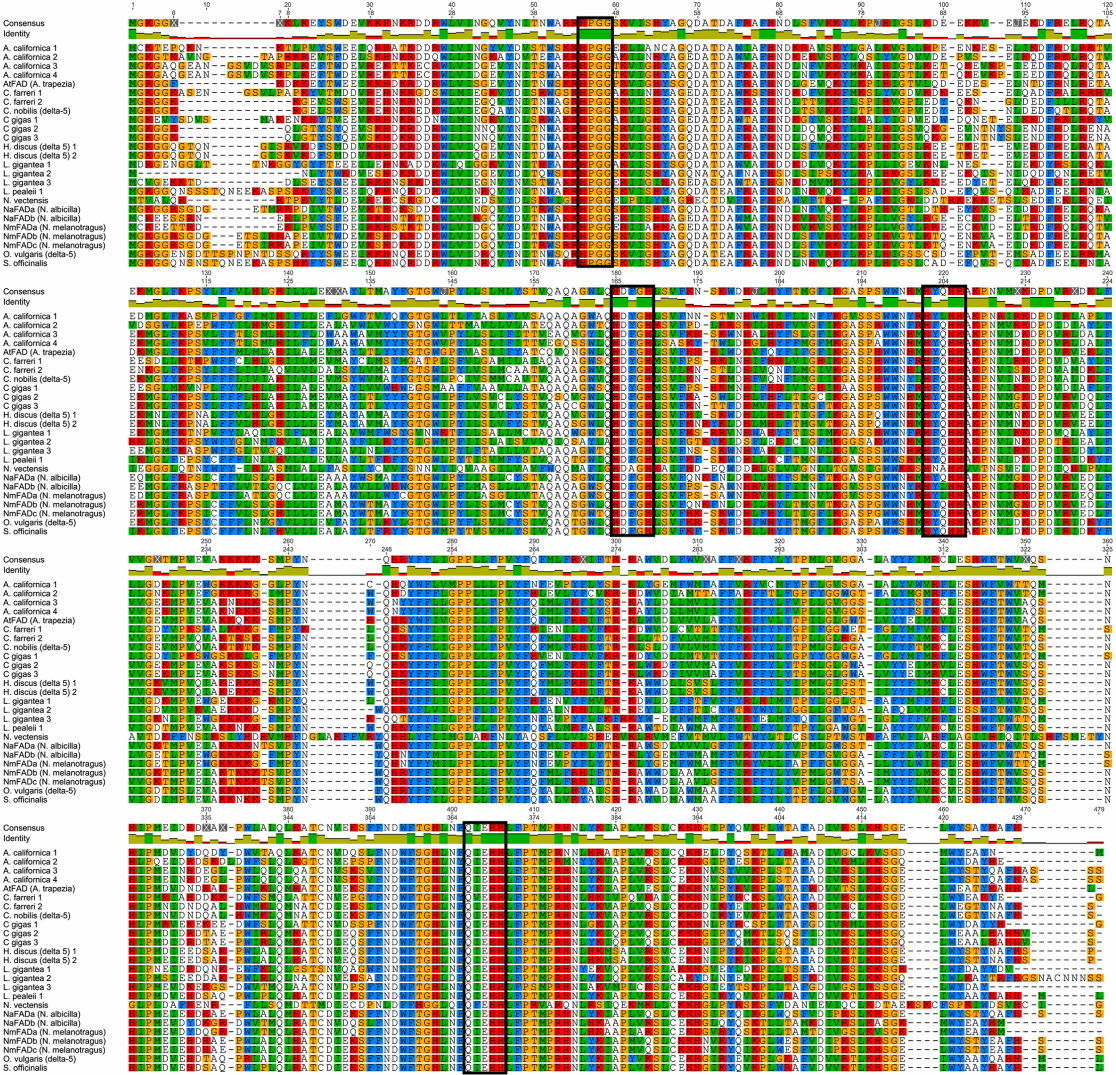
Alignment of Fad protein sequences showing conserved and variable regions. The haeme binding domain (HPGG) and the three histidine boxes (HXXXH, HXXHH and QXXHH) are indicated with a black rectangular outline.

The alignment of 24 mollusc Fad proteins, with the out group protein from *N*. *vectensis*, resulted in an alignment of 483 aa and 57 conserved aa residues being identified (Fig 1). When the *N*. *vectensis* protein sequence was removed, the alignment was considerably shorter (457 aa residues) and 100 conserved aa residues were identified across the 23 protein sequences. Based on the alignment of all identified Fad genes (Fig 1), the haeme binding motif, HPGG and the three histidine boxes, HXXXH, HXXHH and QXXHH were highly conserved across the mollusc species investigated. The first histidine box sequence was highly conserved in aa sequence and consisted of HDF/YGH, where the only difference being the replacement of phenylanine (F) in some species with the structurally similar aa Tyrosine (Y). The second histidine box HXXHH, was also highly conserved and consisted of HY/FQ/LHH. The first difference was the replacement of phenylanine (F) with tyrosine (Y), and the second difference was the replacement of the hydrophilic aa glutamine (Q) with the hydrophobic aa leucine (L). The final histidine box QXXHH box was highly conserved with a sequence of QI/VEHH. Only a single replacement of an isoleucine (I) with the structurally similar aa valine (V) occurred in a *C*. *farreri* protein. All Fad genes had between three to four transmembrane domains.

**Fig 2:**
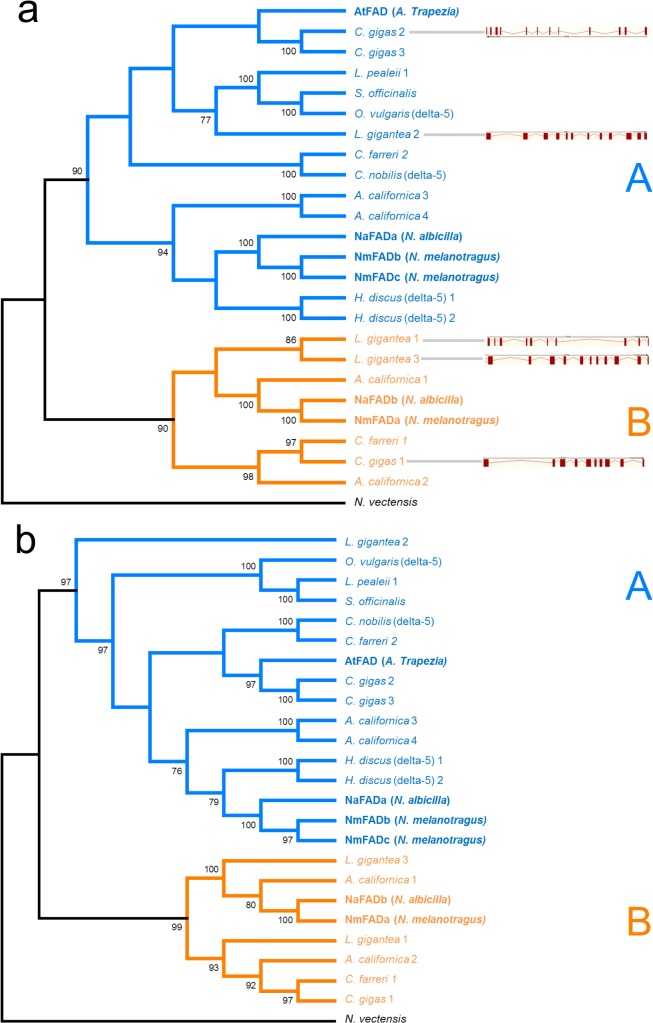
Phylogenetic trees depicting relationships among nucleotide and protein sequences from Fad genes. (a) Maximum Likelihood tree of Fad nucleotide sequences. Bootstrap values are shown next to nodes, values under 75% not reported. Accession numbers: AtFad KR154727, *C*. *gigas* 1 CGI_10016476, *C*. *gigas* 2 CGI_10019765, *C*. *gigas* 3 XM_011415748, *A*. *californica* 1 XM_005096991, *A*. *californica* 2 XM_005093125, *A*. *californica* 3 XM_005090516, *A*. *californica* 4 XM_005090520, *L*. *gigantea* 1 XM_009052983, *L*. *gigantea* 2 XM_009051720, *L*. *gigantea* 3 XM_009046829, *C*. *nobilis* (delta-5) KJ598786, *H*. *discus* (delta-5) 1 GQ470626, *H*. *discus* (delta-5) 2 GQ466197, *O*. *vulgaris* (delta-5) JN120258, *S*. *officinalis* KP260645. Exon-intron structure for *L*. *gigantea* and *C*. *gigas* are presented as gene models with exons (red boxes) and introns (red lines) adjacent to the corresponding species. (b) Maximum Likelihood tree of Fad protein sequences. Bootstrap values are shown next to nodes, and values under 75% not reported. Accession numbers: *C*. *gigas* 1 EKC33620, *C*. *gigas* 2 EKC30965, *C*. *gigas* 3 XP_011414050, *A*. *californica* 1 XP_005097048, *A*. *californica* 2 XP_005093182, *A*. *californica* 3 XP_005090573, *A*. *californica* 4 XP_005090577, *L*. *gigantea* 1 XP_009051231, *L*. *gigantea* 2 XP_009049968, *L*. *gigantea* 3 XP_009045077, *C*. *nobilis* (delta-5) AIC34709, *H*. *discus* (delta-5) 1 ADK38580, *H*. *discus* (delta-5) 2 ADK12703, *O*. *vulgaris* (delta-5) AEK20864, *S*. *officinalis* AKE92955.

Phylogenetic analysis of the full-length ORF sequences identified two well supported clades for the mollusc Fad sequences (Fig 2A). Clade A (coloured in blue) contained all functionally characterised delta-5 desaturases and had sequences from all mollusc groups represented in this study. This clade contained the AtFad, NaFada, NmFadb and NmFadc. The NaFada sequence was sister to the highly similar NmFadb and NmFadc. These three sequences were sister to a group of other gastropod genes that was made up of recently duplicated genes from *A*. *californica* and *H*. *discus*. The AtFad sequence was sister to a pair of recently duplicated *C*. *gigas* genes and this group was sister to all cephalopod Fad sequences from *L*. *pealeii*, *S*. *officinalis* and *O*. *vulgaris*, and a single Fad gene from *L*. *gigantea*. The second distinct clade (clade B, coloured in orange) contained fewer sequences and no functionally characterised Fads. This clade contained the NaFadb and NmFada sequences as well as two bivalve sequences and four other gastropod sequences. The NaFadb and NmFada sequences were sister to each other and occurred in a well resolved clade with *A*. *californica*. These sequences were sister to recently duplicated genes from *L*. *gigantea*. An additional clade could also be observed in clade B containing sequences from *A*. *californica* and the bivalve species, *C*. *gigas* and *C*. *farreri*. The protein tree showed broadly similar topology to the nucleotide tree, containing the same sequences within the two distinct clades (Fig 2B). Within clade A, protein sequences clustered according to taxonomic affinity with the exception of *L*. *gigantea* which was sister to all sequences within clade A. Clade B exhibited largely similar topology to the nucleotide tree with the exception of *L*. *gigantea 1* no longer clustering with its recently duplicated copy, instead clustered with the two bivalve sequences and *A*. *californica 2*.

Exon-intron structure could only be determined for two *C*. *gigas* (*C*. *gigas* 1 and *C*. *gigas* 2) and all three *L*. *gigantea* Fad genes as they have nearly completed genome sequences (Fig 2A). The *A*. *californica* exon-intron structure could not be determined because this genome is too fragmented to reliably confirm the exon-intron structure of gene models. The exon-intron structure of the Fad genes was different between the two clades. Specifically, the two genes found in the clade A that contained functionally characterised delta-5 Fads had 12 exons (*C*. *gigas* 2 and *L*. *gigantea* 2), while the three genes in clade B had 10 (*C*. *gigas* 1 and *L*. *gigantea* 1) and 11 exons (*L*. *gigantea* 3). The two genes from clade B with 10 exons were more closely related than the Fad with 11 exons.

#### Elovls alignment and phylogenetic analysis

**Fig 3:**
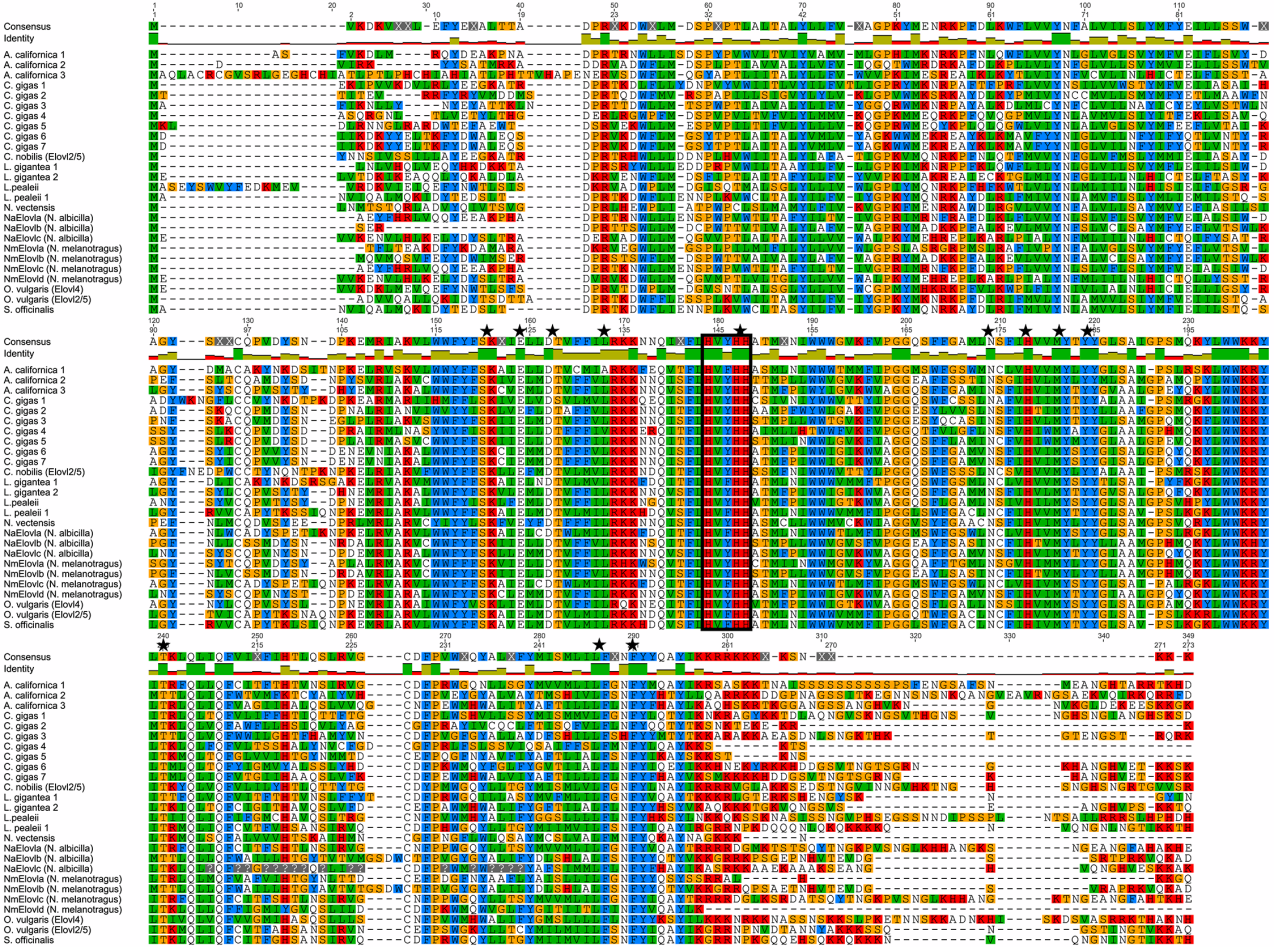
Alignment of Elovl protein sequences showing conserved and variable regions. The histidine box (HXXHH) is indicated with a black rectangular outline. Highly conserved aa residues (K, E, DT, L, HH, N, H, MY, YY, T, LF, F) are designated with the symbol ★.

The alignment of the Elovl protein sequences was 349 aa in length and had 47 conserved aa residues when the *N*. *vectensis* protein sequence was included in the alignment, as well as when removed (Fig 3). The diagnostic histidine box (HXXHH) was highly conserved and consisted of HVF/YHH, where the only difference was the replacement a phenylalanine (F) residue with a tyrosine (Y). Another highly conserved region of 15 aa was identified in the alignment between residues 233–247. Within this region 10 of the 15 aa were identical across all mollusc species. All Elovl genes had between five to seven transmembrane domains. The conserved 17 aa distributed throughout were also conserved, but a few sequences had a single aa substitution. These substitutions occurred in *C*. *gigas* 1 from threonine (T) (position 163 in the Elovl alignment) to a serine (S), *C*. *gigas* 4 leucine (L) (position 168 in the Elovl alignment) to valine (V) and *A*. *californica* 1 leucine (L) (position 168 in the Elovl alignment) to alanine (A).

**Fig 4:**
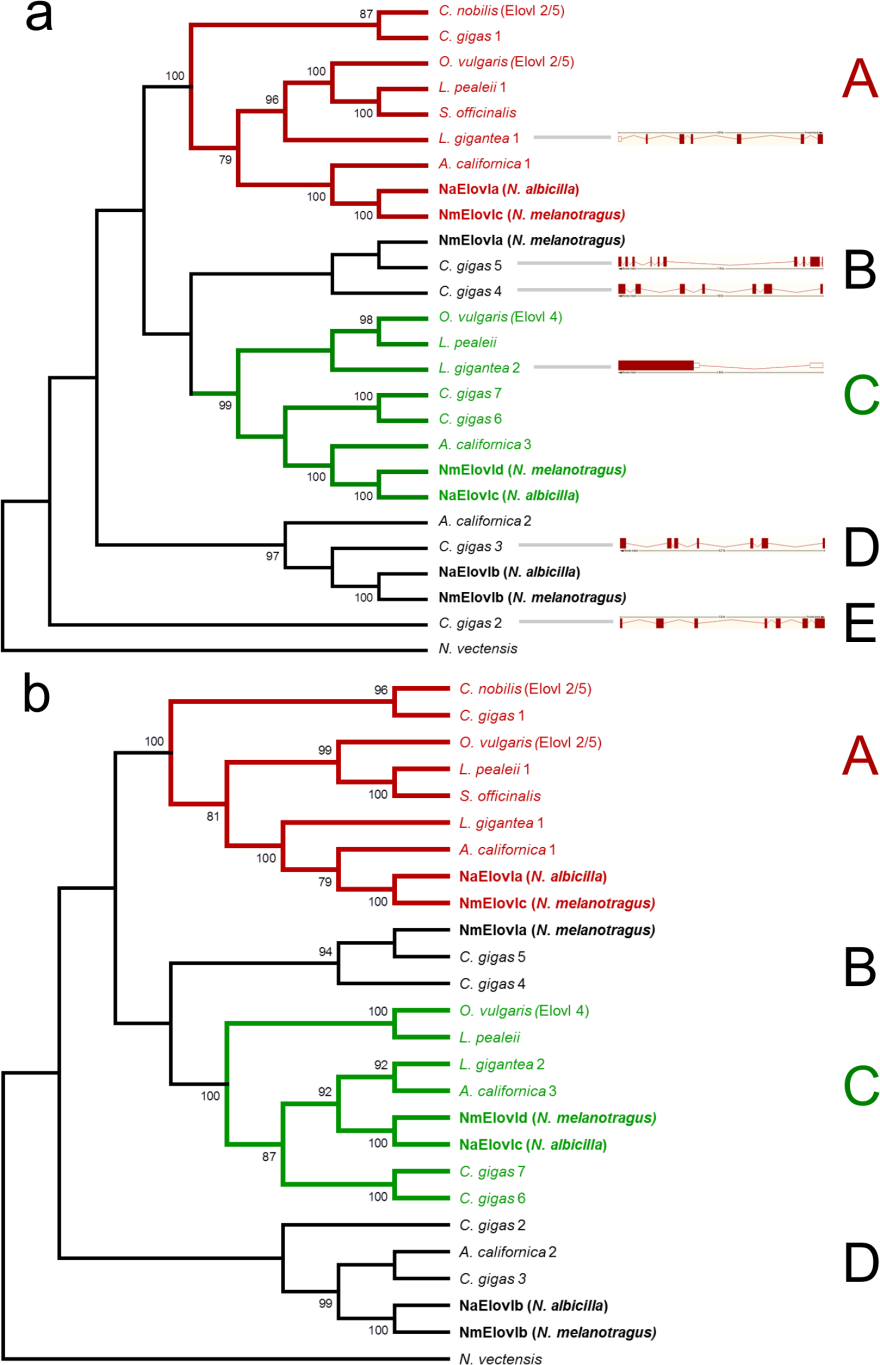
Maximum Likelihood trees of Elovl nucleotide and protein sequences. (a) Phylogenetic tree of Elovl nucleotide sequences. Bootstrap values are shown next to nodes, and values under 75% not reported. Accession numbers: NmElovla KR154728, NmElovlb KR154729, *C*. *gigas* 1 CGI_10028198, *C*. *gigas* 2 CGI_10008431, *C*. *gigas* 3 CGI_10020977, *C*. *gigas* 4 CGI_10012627 *C*. *gigas* 5 CGI_10007566, *C*. *gigas* 6 XM_011452473, *C*. *gigas* 7 XM_011452475, *A*. *californica* 1 XM_005098245, *A*. *californica* 2 XM_005095626, *A*. *californica* 3 XM_005106603, *L*. *gigantea* 1 XM_009047472, *L*. *gigantea* 2 XM_009052848, *C*. *nobilis* (*Elovl2/5*) KF245423, *O*. *vulgaris* (*Elovl4*) KJ590963, *O*. *vulgaris* (*Elovl2/5*) JX020803, *S*. *officinalis KP260646*. Exon-intron structure for *L*. *gigantea* and *C*. *gigas* are presented as gene models with exons (red boxes) and introns (red lines) adjacent to the corresponding species. (b) Phylogenetic tree of Elovl protein sequences. Bootstrap values shown next to nodes, and values under 75% not reported. Accession numbers: *C*. *gigas* 1 CGI_10028198, *C*. *gigas* 2 EKC41251, *C*. *gigas* 3 EKC25061, *C*. *gigas* 4 EKC39214, *C*. *gigas* 5 EKC19804, *C*. *gigas* 6 XP_011450775, *C*. *gigas* 7 XP_011450777, *A*. *californica* 1 XP_005098302, *A*. *californica* 2 XP_005095683, *A*. *californica* 3 XP_005106660, *L*. *gigantea* 1 XP_009045720, *L*. *gigantea* 2 XP_009051096, *C*. *nobilis* (Elovl2/5) AGW22128, *O*. *vulgaris* (Elovl4) AIA58679, *O*. *vulgaris* (Elovl2/5) AFM93779, *S*. *officinalis* AKE92956.

A phylogenetic tree constructed from mollusc Elovl full-length ORF sequences is presented in Fig 4A. Clade A (coloured in red) contained functionally characterised Elovl2/5 sequences from *O*. *vulgaris* and *C*. *nobilis*. Within this clade sequences clustered largely according to taxonomic affinity for bivalves, cephalopods and gastropods, with the exception of *L*. *gigantea*, which clustered with cephalopods. Within clade A, a group composed of bivalve sequences was sister to all cephalopod and gastropod sequences. An additional well resolved clade (clade C) was identified containing the hypothesised *Elovl4* gene from *O*. *vulgaris*. Elovl sequences from all major orders could be found within this clade and again largely clustered according to taxonomic affinity, with the exception of *L*. *gigantea* which clustered weakly with cephalopod sequences. The cephalopod sequences within this clade were sister to bivalve and gastropod sequences. Two additional clades (clades B and D) could be observed and contained no functionally charactersied Elovl sequences. These two clades contained only bivalve and gastropod sequences. The NmElovla sequence (clade B) was closely related to two sequences from *C*. *gigas*, while the NaElovlb and NmElovlb sequences (clade D) were sister to each other and closely related to sequences from *C*. *gigas* and *A*. *californica*. An additional sequence from *C*. *gigas* was also found to be sister to all Elovl sequences. The protein tree in Fig 4B showed similar topology to the nucleotide tree, with the exception of the *C*. *gigas* sequence in clade E now clustering within clade D. The protein tree produced a topology where the sequences in clades A and C clustered according to taxonomic affinity.

The exon-intron structure could only be resolved for four *C*. *gigas* and all *L*. *gigantea* Elovl genes (Fig 4A). The transcripts for *C*. *gigas* 1, 6 and 7 had no exon-intron structure in the current version of the *C*. *gigas* genome. There was no consistent association between exon-intron structure and phylogenetic relatedness for Elovl genes. The single gene (*L*. *gigantea* 1) from clade A, that contained functionally characterised Elovl5s, had seven exons as did the two genes (*C*. *gigas* 2 and 3) from clade E and D respectively with no functionally characterised Elovls. The single gene (*L*. *gigantea* 2) from clade C had two exons. The two genes (*C*. *gigas* 4 and 5) in clade B had seven and 10 exons, respectively.

### PCR Validation

All three primer pairs amplified the expected fragments that were used for PCR validation. The transcripts used for validation were AtFad, NmElovla and NmElovlb. The validation sequence of AtFad (accession: KR154727) was identical to the *de novo* assembled transcript, in protein sequence and only had five synonymous SNPs across the 1293bp ORF, which corresponded to 99.6% nucleotide similarity. The NmElovla (accession: KR154728) and NmElovlb (accession: KR154729) were also identical in protein sequence to the *de novo* assembled transcripts and had high similarity, 99.5% (three synonymous SNPs) to 99.9% (one synonymous SNP) to the ORF of their respective transcripts.

## Discussion

In this study we investigated the distribution and phylogenetic relationship of Fad and Elovl genes, in a number of commercially important orders within the phylum Mollusca. Multiple Fad and Elovl genes were identified in Bivalvia, Gastropoda and Cephalopoda, while the phylogenetic analysis of Fad and Elovl sequences identified multiple well resolved clades in each of these gene families. This finding indicates that both the Fad and Elovl gene families in bivalves and gastropods have undergone at least one round of gene duplication in the last common ancestor of these orders. The novel and newly characterised sequences generated in this study also indicated that lineage specific duplication events have played a prominent role in the evolution and expansion of the Fad gene family in molluscs.

Multiple Fad genes were identified in both *N*. *albicilla* and *N*. *melanotragus*, while only a single Fad was found in *A*. *trapezia*. This pattern, where fewer Fad genes were observed in bivalves when compared to gastropods, was not mirrored across other surveyed species from these orders including those with full genome sequences (i.e. *L*. *gigantea*—3 genes, *A*. *californica*—4 genes, and *C*. *gigas*—3 genes). The presence of genes from both clades, in both bivalves and gastropods, indicates a duplication event in their common ancestor, estimated to be at least 300 million years ago [53]. This duplication event did not occur in cephalopods based on their sequences only occurring in clade A.

Lineage specific duplications of Fad genes were found in both bivalves and gastropods. This lineage specific duplication can be seen in clade A, where *N*. *melanotragus* (NmFadb and NmFadc), *A*. *californica* (*A*. *califonica* 3 and 4), *C*. *gigas* (*C*. *gigas* 2 and 3) and *H*. *discus* (*H*. *discus* (delta-5) 1 and 2), show evidence of recent gene duplications. An additional lineage specific duplication could be observed in clade B for the gastropod species with sequenced genomes, *A*. *californica* 1 and 2 and *L*. *gigantea* 1 and 3. These lineage specific duplication events appear to have increased gene numbers in the gastropod and bivalve lineage compared to the cephalopod lineage.

Duplication of Fad genes has also been observed in vertebrate taxa including mammals [18], birds and reptiles [54] as well as fish [55]. Previous work in vertebrates has found that the distribution of Fads was the result of both ancient and recent duplication events [54]. The ancient duplication predated the split of vertebrates and has resulted in formation of two distinct clades (*Fads1* and *Fads2*), while lineage specific duplication events have led to increased gene copy number in species such as salmon (*Salmo salar*), chicken (*Gallus gallus*) and green anole (*Anolis carolinensis*) [54]. This pattern is largely consistent with our findings where clades A and B are likely to be the result of an ancient duplication event, while the more recent duplications have led to lineage specific expansions of Fad genes in both clades.

The discovery of clade B in the Fad phylogeny represents an interesting finding because it contained only uncharacterised, and potentially novel, proteins. These proteins are of interest for further functional characterisation to determine the specific role of these divergent Fads in the lcPUFA biosynthesis pathway. It may be hypothesised, therefore that if these genes were to have a new function, such as delta 4, 6 or 8 desaturase activity, then all necessary Fad enzymes for omega-3 lcPUFA synthesis would be present. A recent study supports this finding as a desaturase with delta-8 activity has been functionally characterised in the bivalve *C*. *nobilis* [52], allowing for the synthesis of EPA but not DHA. Some marine mollusc species, however, have an increased copy number of Fad genes when compared to the functionally characterised Fad enzymes found in *C*. *nobilis*. This increased copy number found in *C*. *gigas*, along with *L*. *gigantea*, *A*. *californica* and N. *melanotragus*, may therefore provide the additional desaturase activity required for the synthesis of DHA. This is plausible since the capacity for biosynthesis of lcPUFA was demonstrated in the bivalve *C*. *gigas* in previous studies [56, 57].

Multiple Elovl transcripts were identified in both *N*. *albicilla* and *N*. *melanotragus* but no full-length Elovl transcripts were found in the *A*. *trapezia* transcriptome. The observation that gastropods have a greater number of Elovl genes compared to bivalves was not supported by the genomic data, where *C*. *gigas* had the greatest number of Elovl genes (*C*. *gigas*—7 genes, *A*. *californica*—3 genes, *L*. *gigantea*—2 genes). Phylogenetic analysis identified that gene duplication has been a major force in the evolution and diversification of Elovl genes in molluscs. One duplication event in the Elovl gene family is likely to have occurred during the early diversification of molluscs (~ 500 million years ago) as cephalopods, bivalves and gastropods have sequences found in both clades [53]. Lineage specific duplications in *C*. *gigas* were identified in the Elovl phylogeny and are observed in clades B, C and D. This shows that, similar to the Fad gene family, gene duplication has played a major role in the evolution of the Elovl gene family in molluscs.

Duplication and neofunctionalisation have played an important role in the evolution of Elovl genes in various taxonomic groups but remains best studied in fish, where Elovl genes have evolved new functions following an ancient duplication [58]. A similar pattern is seen in our mollusc Elovl phylogeny where two *O*. *vulgaris* homologs, one homolog hypothesised to have Elovl4 activity and the other with Elovl5 activity [10, 23], are found within the different and well resolved clades that form the earliest split in the Elovl phylogeny. In fish, salmon (*S*. *salar*) have an increased number of Elovl genes compared to other species [59]. Again we observed a similar pattern in *C*. *gigas*, which had an increased number of Elovl genes compared to the other mollusc species examined. Functional characterisation of Elovl genes from *C*. *gigas* is needed before we can ascertain whether these duplicated genes have evolved new functions.

The lack of full-length Elovl transcripts and the presence of only a single Fad transcript in *A*. *trapezia* should be viewed with some caution. This is because the *A*. *trapezia* transcriptome was sequenced at a lower depth than the other transcriptomes used for the identification of Fads and Elovls in this study. Given that two bivalve species, *C*. *gigas* and *C*. *farreri*, each have two Fad proteins, and *C*. *gigas* had 7 Elovl proteins, additional Fad and Elovl transcripts may have been identified in *A*. *trapezia* if this transcriptome was sequenced to a greater depth. It is not uncommon that a functional gene is not captured during transcriptome sequencing, in particular if the gene is expressed at low levels or during specific developmental stages [60]. In future studies, more complete transcriptome or genome sequences may improve our ability to resolve the presence and absence of Fad and Elovl genes in these taxa.

In summary, this study has demonstrated that gene duplication has had a large influence on the evolution and distribution of Fad and Elovl genes in mollusc species. The identification of an uncharacterised clade of Fad proteins is of great interest as this may represent a group of Fads with novel functions. Our research also demonstrated that most mollusc species had orthologous genes of characterised Elovl4 and 5 proteins, which suggests that most molluscs can elongate PUFAs to lcPUFAs. Overall, this study has identified a number of uncharacterised Fad and Elovl proteins that require functional characterisation to further illuminate the role of these proteins in lcPUFA synthesis in molluscs.
